# Evaluation of malignancy with thyroid imaging reporting and data system (TI-RADS) in thyroid nodules with persistent nondiagnostic cytology

**DOI:** 10.3906/sag-1811-198

**Published:** 2019-06-18

**Authors:** Hüsniye BAŞER, Oya TOPALOĞLU, Sevgül FAKI, Afra ALKAN, Mustafa Ömer YAZICIOĞLU, Hayriye Tatlı DOĞAN, İbrahim KILINÇ, Reyhan ERSOY, Bekir ÇAKIR

**Affiliations:** 1 Department of Endocrinology and Metabolism, Faculty of Medicine, Yıldırım Beyazıt University, Ankara Turkey; 2 Department of Biostatistics, Faculty of Medicine, Yıldırım Beyazıt University, Ankara Turkey; 3 Department of General Surgery, Atatürk Education and Research Hospital, Ankara Turkey; 4 Department of Pathology, Faculty of Medicine, Yıldırım Beyazıt University, Ankara Turkey

**Keywords:** Nondiagnostic cytology, malignancy, thyroid imaging reporting and data system

## Abstract

**Background/aim:**

We aimed to evaluate the utility of thyroid imaging reporting and data system (TI-RADS) in prediction of malignancy in thyroid nodules with persistent nondiagnostic (ND) cytology.

**Materials and methods:**

A total of 246 thyroid nodules which were surgically removed and had at least two fine-needle aspirations (FNAs) with ND cytology were included in this study. Ultrasonography features and TI-RADS scores were recorded.

**Results:**

Of 246 nodules, 218 (88.6%) had benign and 28 (11.4%) had malignant final histopathology. Frequencies of taller than wide shape, solidity, hypoechogenicity, microcalciﬁcations, and presence of irregular borders were similar between benign and malignant nodules (P > 0.05). The number of nodules categorized as TI-RADS 3, 4a, 4b, and 4c were 12 (4.9%), 53 (21.5%), 104 (42.3%), and 77 (31.3%), respectively. There was not any nodule in TI-RADS 5 category. Malignancy rates of categories 3, 4a, 4b, and 4c were 0%, 13.2%, 9.6%, 14.3%, respectively. No significant differences were detected in TI-RADS categories between benign and malignant nodules (P > 0.05).

**Conclusion:**

In this study, we did not demonstrate any suspicious ultrasound (US) finding predictive for malignancy in thyroid nodules with persistent ND cytology and did not determine any difference between malignant and benign nodules regarding TI-RADS scores. Whereas, we found that thyroid nodules in 4a, 4b, and 4c TI-RADS categories had higher malignancy rates than those previously reported in ND cytology. We think that TI-RADS categories in thyroid nodules with persistent ND cytology can be helpful in treatment decision.

## 1. Introduction

Ultrasound (US)-guided fine-needle aspiration (FNA) is considered as the gold standard, accurate, cost-effective, and safe procedure in evaluation of thyroid nodules (1,2). However, FNA provides extremely specific and sensitive test for pathologic diagnosis, it has also some diagnostic limitations such as nondiagnostic (ND), inadequate, or unsatisfactory results. The estimated rate of malignancy in ND cytology has been reported as 1%–4% in the literature, and in this setting US-guided FNA repetition is recommended (3). The incidence of ND cytology has been reported in a wide range from 3% to 36.4% (2,4–7). A thyroid nodule with ND cytology generally causes diagnostic and therapeutic dilemma. 

Thyroid US is a widely-used, noninvasive method in the evaluation of thyroid nodules (8,9). High risk US features for malignancy are solidity, irregular borders, microcalcifications, marked hypoechogenicity, and more tall than wide shape (8,10,11). The thyroid imaging reporting and data system (TI-RADS) is useful in risk stratification of thyroid nodules by using the number of high risk US features (12). 

TI-RADS has been initially described in 2009 by two independent teams led by Horvath et al. and Park et al. (13,14). However, these systems are found as difficult to apply in daily practice. Recently, Kwak et al. presented a more convenient and simple system for TI-RADS classification based on analysis of suspicious US features (12). Thereafter, several different TI-RADS classifications have been proposed (15–17). 

TI-RADS has been determined as an effective method in malignancy prediction in thyroid lesions with various cytologies (18). In the literature, there has been a few studies evaluating the scoring systems and high risk US features in malignancy prediction of nodules with ND cytology (19,20). The aim of the present study was to assess the utility of high risk US features and TI-RADS in estimation of malignancy in thyroid nodules with persistent (at least two FNAs) ND cytology.

## 2. Materials and methods 

### 2.1. Study population 

The medical records of 2998 patients who underwent thyroidectomy were screened from January 2007 to December 2016, retrospectively. US features, cytology, and histopathology results of 12027 nodules in 2998 patients were recorded. Of 12027 nodules, 2646 (22%) were determined as ND cytology. These 246 nodules with ≥2 ND cytologies were included in the study.

Average time between consecutive FNA procedures was at least 3 month. ND nodules with subsequent other cytologies such as benign, atypia of undetermined significance/follicular lesion of undetermined significance (AUS/FLUS), suspicious for follicular neoplasm (SFN), suspicious for malignancy, and malignant were excluded. 

Besides demographic information, preoperative thyroid function tests (serum thyrotropin (TSH), free triiodothyronine (fT3), free thyroxine (fT4), anti-thyroid peroxidase antibody (anti-TPOAb), anti-thyroglobulin antibody (anti-TGAb)), suspicious US findings, presence of Hashimoto’s thyroiditis (HT), and final histopathology results (malignant/benign) were obtained from medical records. Diagnosis of HT in histopathology was based on the presence of germinal centers, diffuse lymphocytic and plasmacytic infiltration, and enlarged epithelial cells with large nuclei and eosinophilic cytoplasm (Hurthle cells). 

Serum TSH, fT3, fT4, anti-TPOAb, and anti-TgAb levels were measured by chemiluminescence methods (Immulite 2000, Diagnostic Products Corp., Los Angeles, CA, USA and UniCel DXI 800, Beckman Coulter, Brea, CA). The thyroid antibody level over the upper range of normal was evaluated as positive.

Local ethical committee approval was obtained according to the ethical standards of Helsinki declaration (Approval date and number: 22nd March 2017-2017/63).

### 2.2. US examination

Thyroid US was performed using an Esaote color Doppler US (Model 796FDII; MAG Technology Co. Ltd., Yung-Ho City, Taipei, Taiwan) with a superficial probe (Model LA523 13–4, 5.5–12.5 MHz). US examinations were performed by one of three endocrinologists, each with expertise in thyroid US. Suspicious US features (microcalcifications, hypoechogenicity, irregular borders, taller than wide shape, solidity) were noted retrospectively from the US reports by an investigator. The model suggested by Kwak et al. was used for determination of TI-RADS categories (12). These categories were named as follows: category 3, nodules with no suspicious US feature; category 4a, nodules with one suspicious feature; category 4b, nodules with two suspicious features; category 4c, nodules with three or four suspicious features; and finally category 5, nodules with five suspicious US features (12). A TI-RADS score was assigned to each nodule retrospectively by the same investigator evaluating the US reports who was blind to the pathology results.

### 2.3. Fine needle aspiration procedure 

Informed consent was taken from all patients after giving information about the FNA procedure. For the blood thinner users, these medications were stopped temporarily for 5 or more days before the procedure. FNA was carried out with a 27-gauge needle and 20-mL syringe under US guidance (Logic Pro 200 GE and 7.5 MHz probe, Kyunggigo, Korea) by experienced endocrinologists. Each nodule had average 2-3 passes from different sites. Aspirated materials were expelled onto glass slides on-site. FNA samples were air-dried and stained with Giemsa stain. Cytopathologist was not on-site during the procedure. The slides were evaluated off-side. The cytologic results were reported according to the Bethesda classification. Specimens having obscuring blood or insufficient amount of follicular cells, and smears, which were overly thick or air-dried alcohol-fixed, were evaluated as ND.

### 2.4. Statistical analysis

All data were analyzed with SPSS 15.0 software package (SPSS Inc., Chicago, IL, USA). Descriptive statistics were defined as mean±standard deviation (SD) for normally distributed variables, median and range (min-max) for nonnormally distributed variables, and as number of cases (%) for nominal variables. Chi-square test was performed in order to investigate the differences between the groups for categorical variables. The comparisons between groups were performed by Student’s t test and Mann–Whitney U test for parametric and nonparametric variables, respectively. A P value less than 0.05 was accepted as statistically significant.

## 3. Results

Among 246 patients, final histopathological diagnosis was benign in 218 (88.6%) and malignant in 28 (11.4%). However, there were 57 (26.2%) male and 161 (73.8%) female in benign group, 4 (14.3%) male and 24 (85.7%) female patients were present in malignant group (P = 0.171). There was no significant difference in mean age between two groups (P = 0.068). Median TSH was significantly higher in malignant group (P = 0.014). Serum fT3 and fT4 were not statistically different between groups (P = 0.470 and P = 0.400, respectively). Moreover, there was no significant difference in anti-TPOAb and anti-TgAb positivity between benign and malignant groups (P = 0.853 and P = 0.187, respectively). HT was present in 14 (50%) of 28 patients in malignant group and 70 (32.1%) of 218 patients in benign group (P = 0.060) (Table 1).

**Table 1:** Demographic characteristics and baseline laboratory data of patients according to benign and malignant final histopathology.

	Benign(n = 218)	Malignant(n = 28)	P
Age (years)	51.09±11.15	46.64±11.83	0.068
Sex (M/F)	57/161	4/24	0.171
TSH (μIU/mL)	1.00(0.007-9.34)	1.50(0.002-6.15)	0.014
fT3 (pg/mL)	3.20(1.57-6.96)	3.17(2.14-4.39)	0.470
fT4 (ng/dL)	1.14(0.68-2.47)	1.20(0.70-1.67)	0.400
Anti TPO positivity (n,%)	n = 19943 (21.6)	n = 255 (20.0)	0.853
Anti Tg positivity (n,%)	n = 19249 (25.5)	n = 233 (13.0)	0.187
Presence of HT (n,%)	70 (32.1)	14 (50)	0.060

M: male, F: female, fT3: free triiodothyronine, fT4: free thyroxine, anti-TPOAb: anti-thyroid peroxidase antibody, anti-TGAb: anti-thyroglobulin antibody, HT: Hashimoto’s thyroiditis in histopathology. Significant P values are given in bold.

Among 28 nodules with malignant histopathology, 25 (89.3%) were a papillary thyroid carcinoma (PTC), 1 (3.6%) was a follicular thyroid carcinoma (FTC), 1 (3.6%) was a medullary thyroid carcinoma (MTC), and 1 (3.6%) was an undifferentiated thyroid carcinoma (UTC). Of 25 nodules with PTC, 10 (40%) had classical variant, 12 (48%) had follicular variant, 2 (8%) had encapsulated follicular variant, and 1 (4%) had solid variant (Table 2). No significant differences were detected in suspicious US features between benign and malignant nodules (P > 0.05) (Table 3). 

**Table 2:** Distribution of histopathology results in malignant group.

	Malignant (n = 28)
PTC (n,%)	25 (89.3)
Classical variant	10 (40)
Follicular variant	12 (48)
Encapsulated follicular variant	2 (8)
Solid variant	1 (4)
FTC (n,%)	1 (3.6)
MTC (n,%)	1 (3.6)
UTC (n,%)	1 (3.6)

PTC: papillary thyroid carcinoma, FTC: follicular thyroid carcinoma, MTC: medullary thyroid carcinoma, UTC: undifferentiated thyroid carcinoma.

**Table 3:** Comparison of high risk US features and TI-RADS categories between histopathologically benign and malignant groups.

	Benign(n = 218)	Malignant(n = 28)	P
Nodule AP diameter (mm)	12.13±5.77	12.56±5.29	0.704
Nodule T diameter (mm)	15.48±7.64	14.62±7.82	0.583
Taller than wide shape (n,%)	35 (16.1)	5 (17.9)	0.808
Solidity (n,%)	203 (93.1)	28 (100)	0.358
Hypoechogenicity (n,%)	20 (9.2)	3 (10.7)	0.568
Microcalcification (n,%)	65 (29.8)	8 (28.6)	0.892
Irregular border (n,%)	126 (57.8)	18 (64.3)	0.512
TI-RADS (n, %)			
TI-RADS 3	12 (5.5)	0	0.203
TI-RADS 4a	46 (21.1)	7 (25.0)	0.637
TI-RADS 4b	94 (43.1)	10 (35.7)	0.455
TI-RADS 4c	66 (30.3)	11 (39.3)	0.333
TI-RADS 5	0	0	-

AP: anteroposterior, T: transverse, TI-RADS: thyroid imaging reporting and data system.

Of 246 nodules with ND cytology, 12 (4.9%) were assigned as TI-RADS 3, 53 (21.5%) as TI-RADS 4a, 104 (42.3%) as TI-RADS 4b, and 77 (31.3%) as TI-RADS 4c. Thyroid nodule with TI-RADS 5 category was not detected. No malignancies were detected in the group of nodules assigned a TI-RADS 3 category. The malignancy rates of TI-RADS category 4a, 4b, and 4c nodules were 13.2% (7 of 53 nodules), 9.6% (10 of 104 nodules), and 14.3% (11 of 77 nodules), respectively (Table 4). No significant differences were detected in TI-RADS categories between benign and malignant nodules (P > 0.05) (Table 3).

**Table 4:** Histopathology results of nodules according to TI-RADS categories.

	TI-RADS 3n = 12	TI-RADS 4an = 53	TI-RADS 4bn = 104	TI-RADS 4cn = 77	Test statistics	p
Benign (n,%)	12 (100)	46 (86.8)	94 (90.4)	66 (85.7)	2.682	0.443
Malignant (n,%)	0	7 (13.2)	10 (9.6)	11 (14.3)		

TI-RADS: thyroid imaging reporting and data system.

## 4. Discussion

The Bethesda system was suggested in 2009 to standardize the terminology for interpreting cytology reports among pathologists and laboratories, and to eliminate diagnostic and therapeutic dilemmas. Although a small number of cytologies represent Bethesda Category I (ND), it is the most important limitation of thyroid FNA. Furthermore, ND cytology results substantially affect the management of nodules. Although, Bethesda system recommended that ND cytology should not exceed 10% of thyroid FNA, a wide range of its usage (up to 36.4%) has been reported in the literature (3,7,21–26). Due to the 1%–4% low risk of malignancy, the Bethesda system recommends repetition of US-guided FNA in nodules with ND cytologic results. This malignancy rate is similar to the 0%–3% malignancy rate of benign cytology for which clinical follow-up is recommended by the system (3). In cases with ND cytology, actual management may depend on other factors such as clinical and sonographic features of nodules. Yet, there are currently no sufficient data for the management of the nodules with persistent ND FNAs. Due to the approximately 10% approved risk of malignancy for persistent ND cytologies, surgery may be considered in these nodules (27). In the present study, the risk of malignancy was found as 11.4% (28 of 246). In our institution, 2646 (22%) of 12027 thyroid nodules had ND cytology. Of 2646 ND nodules, 246 (9.3%) had persistent ND cytologies. The high rate of ND cytology in our institiution can be associated wih different operators performing the procedure, using different aspiration techniques in the evaluation of fine-needle cytology, and different cytologists evaluating the cytologies. 

Suspicious US features associated with an increased risk of malignancy are hypoechoic echogenicity, presence of irregular borders, microcalcifications, solid pattern, and taller than wide shape (28). We did not find any difference in high risk US features between malignant and benign groups in the present study. Furthermore, we did not demonstrate any suspicious US finding predictive for malignancy in thyroid nodules with persistent ND cytology. Our study population included only the operated patients with ND cytology. In these patients, we decided surgery according to the presence of suspicious US features, which might cause statistically insignificant results between groups. 

Several studies are present in the literature evaluating the TI-RADS in ND cytology (19,20). Recently, Moon et al. evaluated the US features of 548 ND nodules and classified these nodules according to TI-RADS (19). In their study, the malignancy risks for categories 3 and 4a nodules were reported as 0.8% and 1.8%, respectively. Moreover, the same authors found the malignancy rates of categories 4b, 4c, and 5 nodules as 6.1%, 14.4%, and 31%, respectively (19). Yoon et al. stratified the nodules with ND cytology according to TI-RADS before and after application of the Bethesda system (20). They reported the malignancy risks in category 3, 4a, 4b, 4c, and 5 as 1.8%, 5.7%, 4.1%, 29.8%, and 16.7% for the pre-Bethesda period, and 1.6%, 3.0%, 7.1%, 16.3%, and 25.0% for the post-Bethesda period, respectively. They concluded that similar malignancy rates were found in the pre- and post-Bethesda periods (20). In the present study, no malignancies were detected in the group of nodules with TI-RADS category 3. Our malignancy risks of 4a, 4b, and 4c categories were 13.2%, 9.6%, and 14.3%, respectively. Our study is different than those of Moon et al. and Yoon et al. according to the study population. However, Moon et al. studied all of ND nodules including the ones under follow-up, Yoon et al. analyzed ND nodules on which surgery had been performed, nodules at follow-up US guided FNA that showed no significant change, and also nodules with definitive benign or malignant cytologic results at follow-up US-guided FNA (19,20). In our study, we assessed the thyroid nodules with persistent ND cytology, which were surgically removed. Nodules with consecutive benign or malignant cytology, and nodules at follow-up without operation were excluded. Thus, 100% of nodules in our study have surgically proven histologies. 

In the present study, no malignancies were detected in the group of nodules assigned a TI-RADS category of 3. Due to the low risk of malignancy, it has been reported that US follow-up can be considered for thyroid nodules with ND cytology if high risk US features are not present (19). Furthermore, this study demonstrated that the malignancy rates of thyroid nodules with categories 4a, 4b, and 4c were 13.2%, 9.6% and 14.3%, respectively. Thus, in these cases, repetition of US-guided FNA should be considered (19). In the present study, malignancy risks of nodules with categories 4a and 4c are near 15% which is the cut-off for recommending surgery, so surgery can be considered for these nodules (3). 

Thyrotrophin is a major regulator of thyroid hormone production by acting on thyroid follicular cells. Furthermore, TSH consists of a vital role in normal thyroid function and thyroid diseases (29,30). Recently, it has been reported that TSH has a role in thyroid malignancy development and progression, and acts like a growth factor (31,32). Elevated TSH level was found as a risk factor for thyroid malignancy (33–35). Boelaert et al. demonstrated a higher malignancy rate in patients with TSH levels between 1.0–1.7 mU/L compared to TSH levels <0.4 mU/L, and furthermore a particularly higher rate was found in patients with TSH levels more than 1.8 mU/L (35). In the present study, mean TSH level in the malignant group was found higher as compared to the benign group. 

The follicular variant of PTC is the most common subtype after classical variant, constituting between 10% and 15% of all PTC cases (36,37). However, FNA is a sensitive procedure, its reported sensitivity is significantly low in follicular variant of PTC when compared to conventional PTC (38,39). In the present study, 89.3% (25 of 28 nodules) of patients with malignancy had PTC, of which 48% was a follicular variant. 

Our study had several limitations. Firstly, it has a retrospective study design, which causes difficulty in evaluating some factors. Secondly, we evaluated previous US features only in the patients who underwent thyroid surgery. However, in clinical practice, majority of the patients with ND cytology are followed-up without operation. In this study, only 9.3% of patients with ND cytology were operated, most of our patients were going to follow-up. Thus, our results should not be generalized to all of nodules with ND cytology. Thirdly, the nodules with ND cytology, which were operated also had suspicious US features. As a result, our study population had a small number of nodules with TI-RADS category 3. Fourthly, the interval between consecutive US-guided FNAs was not evaluated in our study. In the Bethesda system, for reparation of atypia, although FNA is recommended after 3 months, the optimal interval between two procedures is not described clearly. Some authors recommend further FNA within the following 6–12 months (3,23,40). In our institution, repeat FNA was generally performed at least 3 months after the previous procedure. 

In conclusion, we found malignancy rate as 11.4% in thyroid nodules with persistent ND cytology. Furthermore, we did not demonstrate any suspicious US finding predictive for malignancy in thyroid nodules with persistent ND cytology and did not determine any difference between malignant and benign nodules in TI-RADS scores. In the evaluation of TI-RADS categories for all nodules with persistent ND cytologies, no malignancy was detected in TI-RADS category 3 but malignancy rate was increased to 14.3% in TI-RADS 4 category. However, there are currently no sufficient data for the management of the nodules with persistent ND, TI-RADS categories can be helpful for the clinician in the treatment decision of these nodules.
